# Pulmonary Hypertension in Patients with Chronic Kidney Disease on Dialysis and without Dialysis: Results of the PEPPER-Study

**DOI:** 10.1371/journal.pone.0035310

**Published:** 2012-04-18

**Authors:** Stefan Pabst, Christoph Hammerstingl, Felix Hundt, Thomas Gerhardt, Christian Grohé, Georg Nickenig, Rainer Woitas, Dirk Skowasch

**Affiliations:** 1 The Department of Internal Medicine II – Cardiology/Pneumology, University of Bonn, Bonn, Germany; 2 The Department of Internal Medicine I - Nephrology, University of Bonn, Bonn, Germany; 3 Praxis für Nieren- und Hochdruckkrankheiten Bonn, Bonn, Germany; 4 The Lungenklinik Berlin-Buch, Berlin, Germany; University of Washington, United States of America

## Abstract

Pulmonary hypertension (PH) is common in patients with dialysis-dependent chronic kidney disease and is an independent predictor of mortality. However, specific hemodynamics of the pulmonary circulation, changes induced by hemodialysis and characterization into pre- or postcapillary PH have not been evaluated in patients with chronic kidney disease. We assessed consecutive patients with end-stage chronic kidney disease in WHO FC≥II with dyspnea unexplained by other causes on hemodialysis (group 1, n = 31) or without dialysis (group 2, n = 31) using right heart catheterization (RHC). In group 1, RHC was performed before and after dialysis. In end-stage chronic kidney disease, prevalence of precapillary PH was 13% (4/31), and postcapillary PH was discovered in 65% (20/31). All four cases of precapillary PH were unmasked after dialysis. In group 2, two cases of precapillary PH were detected (6%), and postcapillary PH was diagnosed in 22 cases (71%). This is the first study examining a large cohort of patients with chronic kidney disease invasively by RHC for the prevalence of PH. The prevalence of precapillary PH was 13% in patients with end-stage kidney disease. That suggests careful screening for precapillary PH in this selected patient population. RHC should be performed after hemodialysis.

## Introduction

The prevalence of chronic kidney disease (CKD) in the developed world is 13% [1] and is recognized as a condition that elevates the risk of cardiovascular complications as well as kidney failure and other complications. End-stage kidney disease (ESKD) substantially increases the risk of death, cardiovascular disease, and use of specialized health care. In this context, pulmonary hypertension (PH) has been reported in patients with ESKD maintained on long-term hemodialysis. Based on echocardiographic studies, the prevalence of PH in these patient populations is estimated to be around 17–56% [2]–[7], and PH is an independent predictor of mortality in such patients [6], [7]. However, these studies lack invasive hemodynamic data and thus cannot discriminate between pre- and postcapillary PH in unselected patients with or without symptoms.

PH is a hemodynamic and pathophysiological state found in a range of clinical conditions and is characterized by an increase in mean pulmonary arterial pressure (mPAP ≥25 mmHg); precapillary PH is defined by the additional criterion of a pulmonary arterial wedge pressure (PCWP) ≤15 mmHg [8]. The different forms of PH have been classified into five clinical groups with specific characteristics [8], [9]. Group 1 consists of the major forms of pulmonary arterial hypertension (PAH: idiopathic, heritable and associated with connective tissue disease and congenital heart disease etc.). A diagnosis of PAH requires the exclusion of all other causes of PH, and specific treatments are available. Group 2 describes PH due to left heart disease including diastolic dysfunction, Group 3 PH due to lung diseases and/or hypoxia and Group 4 is chronic thromboembolic pulmonary hypertension (CTEPH). Group 5 consists of PH with unclear and/or multifactorial mechanisms including “chronic renal failure on dialysis" [8], [9]. The pathogenesis of PAH is poorly understood, and the associated conditions that result in PAH are heterogenous and seemingly unrelated.

The purpose of the PEPPER-study (“prevalence of precapillary pulmonary arterial hypertension in patients with end-stage renal disease") was to assess the specific hemodynamics in CKD patients with otherwise unexplained dyspnea on hemodialysis and in those without dialysis, to elucidate possible risk factors contributing to PH, and to evaluate hemodynamic changes induced by hemodialysis – by use of right heart catheterization (RHC), the “gold standard" method for the diagnosis and characterization in pre- and postcapillary PH.

## Methods

### Patients

**Figure 1:**
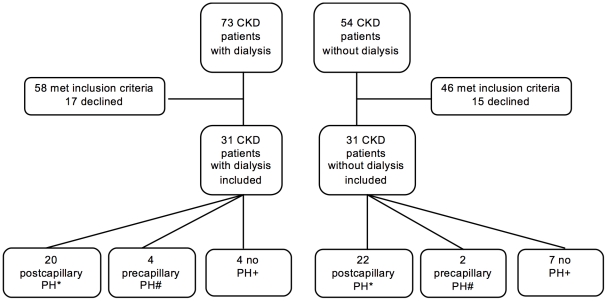
Enrolment of Patients. CKD: chronic kidney disease; Pulmonary hypertension (PH): mean pulmonary arterial pressure (mPAP) ≥25 mmHg; *postcapillary PH: mPAP ≥25 mmHg and pulmonary capillary wedge pressure (PCWP) ≥15 mmHg; #precapillary PH: mPAP ≥25 mmHg and PCWP <15 mmHg, +no PH: mPAP <25 mmHg.

This was a prospective, single center study conducted at the University of Bonn, Germany. Local ethics committee approval was obtained prior to the inclusion of any patient in the study (Ethics committee, University of Bonn, Germany, 061/09) and the study was conducted according to the Declaration of Helsinki. Written informed consent was obtained from all participants involved in our study. Consecutive patients with severe CKD stage 4 or 5 [10] attending the clinic for regular treatment were assessed for enrollment suitability using defined inclusion and exclusion criteria. Within the one year ESKD patients with dialysis were recruited and compared to patients with CKD without dialysis. The study started in November 2009 and ended in October 2010 after 62 patients (31 patients in each group) were included. Detailed information is given in figure 1. Inclusion criteria were: adults ≥18 years old, stage 4 or 5 CKD (defined as serum creatinine ≥200 µmol/l [2.26 mg/dl] or glomerular filtration rate [GFR] ≤30 ml/min/1.73 m^2^ assessed by MDRD4-formula [11], [12] for a time span ≥1 year), on hemodialysis or without hemodialysis treatment, and in World Health Organization functional class (WHO FC) ≥II with dyspnea unexplained by other causes. Exclusion criteria were: uncontrolled arterial hypertension (defined as mean blood pressure before entry into the study ≥160/100 mmHg), current malignant diseases, pregnancy, left ventricular ejection fraction (LVEF) <50%, mitral or aortic regurgitation >grade 2, aortic or mitral surface <1.5 cm^2^, myocarditis, endocarditis, pericarditis, severe anemia (hemoglobin concentration <10 g/dl), severe chronic obstructive pulmonary disease (COPD) defined by FEV_1_ <60% predicted, lung fibrosis, and known PAH medication with prostanoids, endothelin receptor antagonists, or phosphodiesterase-5 inhibitors. The following assessments were undertaken in all patients: medical history (including exact data concerning immunosuppressive medication, other medication and duration of renal insufficiency before starting dialysis and the time under dialysis as well as the daily amount of residual diuresis); clinical examination, including height, (dry-)weight, blood pressure; standard 12-lead-electrocardiography (ECG); transthoracic echocardiography (TTE); lung function testing (bodyplethysmography); laboratory investigations including blood count, and potassium, sodium, aspartate aminotrasferase/alanine aminotransferase (AST/ALT), creatinine and urea levels.

### Right heart catheterization

All patients underwent RHC. RHC in CKD patients on dialysis was performed before and after dialysis, if PH was confirmed with the first RHC. As shown in table 1 PH was defined as mean pulmonary arterial pressure (mPAP) ≥25 mmHg regardless of the pulmonary capillary wedge pressure (PCWP). If mPAP was ≥25 mmHg and PCWP was ≤15 mmHg, the diagnosis of precapillary PH was made. In case of precapillary PH, a complete work-up (including chest computertomography-scan, ventilation-perfusion-scan, sleep apnea screening, ultrasound of the liver and laboratory testing) was performed to verify or exclude PAH. Vasoreactivity testing (inhaled iloprost 5 µg; iNeb, Philips Healthcare, Eindhoven, Netherlands) was performed in case of precapillary PH/PAH. Positive vasoreactivity was defined as a decrease of mPAP ≥10 mmHg to reach ≤40 mmHg with a stable cardiac index (CI) [8]. Cardiac index was measured by direct Fick method.

**Table 1:** Distinction of PH, precapillary PH and postcapillary PH [8].

no PH	mPAP <25 mmHg
PH	mPAP ≥25 mmHg
precapillary PH	mPAP ≥25 mmHg and PCWP <15 mmHg
postcapillary PH	mPAP ≥25 mmHg and PCWP ≥15 mmHg

### Statistical analysis

The German version of SPSS V17.0 (IBM, Munich, Germany) was used as a database and for statistical analysis. Data are expressed as mean ± standard deviation and as a percentage for categorial parameters. Differences between groups were compared with Student's t-test and Mann-Witney-U test, as applicable. Chi-square test was used to estimate the occurrence of categorical variables. Two-tailed bivariate correlations were determined by the Pearson's coefficient. Statistical significance was set at p<0.05.

## Results

### Study population

From November 2009 to October 2010, consecutive patients with severe CKD stage 4 or 5 and in WHO FC ≥II with dyspnea unexplained by other causes were screened by transthoracic echocardiography (TTE) for study participation (Fig. 1).

In all, 62 patients met the inclusion criteria and agreed to participate in the study. The demographics and characteristics of the per-protocol population comprising 31 CKD patients on chronic hemodialysis treatment (group 1) and 31 patients with CKD (group 2) not on dialysis are presented in Table 2. We observed significant differences in age (the mean age of the dialysis cohort was 8 years lower than the nondialysis group), body mass index (BMI was lower in the dialysis group), median time to enrollment after manifestation of CKD (4.9 years in the dialysis group vs. 1.5 years in the non-dialysis group), and use of calcium channel blockers (increased in the dialysis group).

**Table 2:** CHARACTERISTICS OF PEPPER PARTICIPANTS.

Characteristics	Dialysis patients	Patients with CKD (serum creatinine ≥200 µmol/l) without dialysis	p-value
	n = 31	n = 31	
**Age at examination (yrs)**	65.3±7.4	73.6±9.5	**<0.001**
**Gender (% female)**	35	48	0.303
**BMI (kg/m^2^)**	24.0±3.5 (post-dialysis)	26.8±5.17	**0.015**
**GFR (re-expressed MDRD ml/min)**	n.a.	21.94±4.37	n.a.
**CKD cause**			0.152
diabetes mellitus	10 (32%)	13 (42%)	0.430
arterial hypertension	4 (13%)	7 (23%)	0.319
Glomerulonephritis	3 (10%)	5 (16%)	0.449
ADPKD	4 (13%)	0 (0%)	**0.039**
others (including unclear)	10 (32%)	6 (19%)	0.246
**Median time to enrollment (years)**			
from first diagnosis of CKD	4.9±3.8	1.5±12.2	**<0.001**
from first dialysis	3.8±3.5	n.a.	n.a.
**Comorbid conditions**			
Cerebrovascular disease (CVD)	4 (13%)	8 (26%)	0.199
MI in medical history	4 (13%)	6 (19%)	0.490
CAD	10 (32%)	13 (42%)	0.430
PCI	8 (26%)	10 (32%)	0.258
CABG	2 (7%)	3 (10%)	0.416
Neoplasm in medical history	2 (7%)	0 (0%)	0.151
PAD	4 (13%)	4 (13%)	1.0
Atrial fibrillation	13 (42%)	19 (61%)	0.127
COPD (I–II)	9 (29%)	9 (29%)	1.0
Diabetes mellitus	12 (39%)	12 (39%)	1.0
Insulin use	8 (26%)	8 (26%)	1.0
Arterial hypertension	17 (55%)	19 (61%)	0.203
Smoking (actual and former)	19 (61%)	19 (61%)	1.0
Hyperlipidemia	23 (75%)	24 (77%)	0.767
**Medication**			
Beta blockade	21 (68%)	19 (61%)	0.596
Calcium channel blockade	11 (35%)	3 (10%)	**0.015**
ACE inhibitor	13 (42%)	13 (42%)	1.0
AT-1 blockade	4 (16%)	4 (13%)	1.0
Statin	23 (75%)	24 (77%)	0.767
**Dyspnea WHO grade II/III/IV**	22/7/2 (71%/23%/6%)	19/10/2 (61%/32%/7%)	0.586

BMI: body mass index; GFR: glomerular filtration rate; CKD: chronic kidney disease; ADPKD: autosomal dominant polycystic kidney disease; MI: myocardial infarction; CAD: coronary artery disease; PCI: percutaneous coronary intervention; CABG: coronary artery bypass graft; PAD: peripheral artery disease; COPD: chronic obstructive pulmonary disease; AT-1: angiotensin 1.

### Right heart catheterization

All 62 patients underwent RHC; data are given in Table 3.

**Table 3:** HAEMODYNAMIC MEASUREMENTS IN TTE AND RHC.

	All dialysis patients	Dialysis patients with PH		p-value	CKD patients without dialysis	p-value	p-value	p-value
	before dialysis	before dialysis	after dialysis	dialysis before vs. dialysis after		dialysis before vs. no dialysis	patients with PH: dialysis *before* vs. no dialysis	patients with PH: dialysis *after* vs. no dialysis
	n = 31	n = 25	n = 25		n = 31			
**TTE**								
PAP systolic (mmHg)	43±16	44±16	37±13	**<0.001**	43±13	0.908	0.679	0.122
No. of patients PAP systolic ≥30 mmHg	22 (71%)	18 (72%)	15 (60%)	0.370	24 (77%)	0.562	0.642	0.159
LVEF (%)	59±12	61±12	60±11	0.870	56±6	0.170	0.057	0.121
Pericardial effusion	0	0	0	1.0	0	1.0	1.0	1.0
**RHC**								
PAP systolic (mmHg)	56±21	62±18	55±17	**<0.001**	52±15	0.324	**0.019**	0.456
PAP diastolic (mmHg)	27±13	30±11	26±10	**<0.001**	25±9	0.543	0.053	0.559
PAP mean (mmHg)	38±15	42±13	36±12	**<0.001**	35±11	0.315	**0.025**	0.573
PCWP (mmHg)	23±9	25±8	20±6	**<0.001**	22±8	0.917	0.263	0.255
PH	25 (81%)	25 (100%)	24 (96%)	0.327	24 (77%)	0.755	**0.011**	**0.048**
precapillary PH	0 (0%)	0 (0%)	4 (16%)	**0.043**	2 (6%)	0.151	0.196	0.251
postcapillary PH	25 (81%)	25 (100%)	20 (80%)	**0.012**	22 (71%)	0.374	**0.003**	0.438
RAP (mmHg)	14±8	13±9	13±9	1.0	13±6	0.788	0.167	0.167
PVR (dyn · sec · cm^−5^)	345±360	403±378	400±398	0.716	325±340	0.828	0.422	0.451
CI (l/min/m^2^)	2.43±0.79	2.28±0.65	2.28±0.76	0.700	1.94±0.53	**0.005**	**0.033**	**0.028**
TPG (mmHg)	15±10	18±10	17±11	0.142	12±9	0.227	0.057	0.079

CI: cardiac index; CKD: chronic kidney disease: serum creatinine ≥200 µmol/l; LVEF: left ventricular ejection fraction; PAP: pulmonary artery pressure; PCWP: pulmonary capillary wedge pressure; PH: pulmonary hypertension (PAP mean ≥25 mmHg); PVR: pulmonary vascular resistance; RAP: right arterial pressure; RHC: right heart catheterization; TPG: transpulmonary gradient; TTE: transthoracic echocardiography.

In group 1, RHC was performed before and after dialysis, if mPAP was ≥25 mmHg before dialysis (n = 25). If mPAP was determined as <25 mmHg by RHC before dialysis, PH/PAH was excluded, and patients did not undergo a second RHC (n = 6). PH was observed in 25/31 (81%) patients in the dialysis group (before dialysis) versus 22/31 (71%) in the nondialysis cohort. After dialysis in group 1, prevalence of PH was 24/31 (77%, 20/31 postcapillary PH, 4/31 precapillary PH). There was a significant decrease of mPAP and PCWP after dialysis (mPAP from 62±18 to 55±17 mmHg; and PCWP from 25±8 to 20±6 mmHg). All four cases of precapillary PH were identified only by the RHC performed after dialysis treatment; none of the four patients was vasoreactive to inhaled iloprost.

In nondialysis patients (group 2), postcapillary PH was diagnosed in 22/31 cases (71%); precapillary PH without vasoreactivity was found in 2/31 cases (6%). Hemodynamic data were similar to the dialysis group (Table 3); only the higher CI in the dialysis group reached significance.

### Prevalence of precapillary and postcapillary PH

The clinical and hemodynamic profiles of the patients with precapillary PH are displayed in Table 4. Further diagnostic workup according to clinical guidelines [8], including chest CT-scan, ventilation-perfusion-scan, sleep apnea screening, ultrasound of the liver and laboratory testing, confirmed precapillary PH due to hemodialysis (Dana Point Group 5 [9]) in three patients, and excluded PAH and diagnosed PH due to lung diseases and/or hypoxia in three further cases (Dana Point Group 3 [9]; two patients with mild PH and COPD, one patient with mild PH and sleep disordered breathing). In a total of 3/31 CKD patients on dialysis precapillary PH that was not explained by the PH workup (i.e. Dana Point group 1 or 5) was diagnosed, whereas precapillary PH (Dana Point group 1 or 5) was not found in nondialysis CKD patients. Thus, prevalence of precapillary PH (Dana Point group 1 or 5) was 10% in CKD patients on dialysis. There were no further clinically remarkable differences between the patients with precapillary compared to postcapillary PH or between patients with or without PH.

**Table 4:** CHARACTERIZATION OF PATIENTS WITH PRECAPILLARY PH.

Patient	Dialysis/Non-Dialysis	Gender	Age	PAPmean (mmHg)	PCWP (mmHg)	TPG (mmHg)	CI (l/min/m^2^)	PVR (dyn · sec · cm^−5^)	RAP (mmHg)	putative cause for precapillary PH
**1**	Dialysis	m	75	40	12	28	1.3	861	6	dialysis
**2**	Dialysis	m	79	32	14	18	2.8	282	11	severe sleep apnea
**3**	Dialysis	f	70	30	12	18	1.9	497	11	dialysis
**4**	Dialysis	m	58	56	13	33	0.9	1911	20	dialysis
**5**	Non-Dialysis	f	58	29	12	17	1.8	400	6	COPD GOLD II
**6**	Non-Dialysis	m	74	41	13	28	2.8	373	7	COPD GOLD II

Haemodynamic data are from post-dialysis (in dialysis patients).

m: male; f: female; COPD: chronic obstructive pulmonary disease; GOLD stages: global initiative for chronic obstructive lung disease.

## Discussion

We present the results of the first prospective study evaluating the prevalence of precapillary PH by use of RHC in a large cohort of patients with CKD on dialysis or without dialysis. In this symptomatic cohort with dyspnea WHO FC ≥II, the prevalence of precapillary PH (Dana Point group 1 or 5) was found to be 10% in the examined CKD patients requiring renal replacement therapy, whereas no cases of precapillary PH were detected in nondialysis CKD patients. In contrast, the prevalence of PH in CKD patients on or without dialysis was similar (77 vs. 71%, respectively) and considerably higher than previously reported (56 vs. 39% [3]; 44 vs. 32% [6]). The reason for the higher prevalence in our study could be due to the high risk nature of our cohort which included only patients with dyspnea in WHO FC ≥II, whereas other studies also included asymptomatic patients [2]–[7]. Another strength of our study is the use of invasive methodology (RHC), considered a requirement and the “gold standard" for the differential diagnosis between pre- and postcapillary PH and assessment of hemodymic impairment [8], for all study participants. Previous studies used only echocardiographic estimation of systolic PAP for PH diagnosis [2]–[7].

Since the prevalence of precapillary PH in our participants dramatically exceeds the prevalence of PAH in the general population of 15–50 per million adult population [8], [10], end-stage kidney disease or dialysis itself may be a trigger for the development of precapillary PH in a predisposed patient, analogous to connective tissue disease, HIV, or portal hypertension. Hormonal and metabolic disturbances associated with CKD requiring dialysis might lead to pulmonary vascular constriction [8]. There are several pathogenetic mechanisms which may contribute to the development of precapillary PH in patients undergoing long-term dialysis, including impaired endothelial function [13], decreased bioavailability of nitric oxide (NO) [14], [15], and increased levels of endothelin (ET)-1 [16]–[19]. PAP may also be increased by high cardiac output resulting from the arteriovenous access and/or concomitant renal anemia, as well as from fluid overload [9], [20], [21]. In addition, diastolic and systolic left heart dysfunctions are frequent in this setting [9] as also indicated by the high rate of postcapillary PH in this study. Due to the fact that we have excluded patients with reduced left ventricular ejection fraction the main diagnosis in our cohort probably is diastolic dysfunction.

In some patients, it may be difficult to distinguish between a diagnosis of precapillary PH and heart failure with preserved ejection fraction/diastolic dysfunction. In the present study, in CKD patients on dialysis, precapillary PH initially masked by fluid overload was unmasked by dialysis in 4/25 cases of primarily postcapillary PH. However, one may argue the opposite i.e. masking of postcapillary PH by fluid withdrawal. In particular, exercise hemodynamics or volume challenge have been proposed as means of identifying LV dysfunction, but these diagnostic tools require further standardization and can often not be applied to dialysis patients [8]. Even so, an elevated transpulmonary pressure gradient (TPG; mPAP minus PCWP) >12 mmHg is suggestive of intrinsic changes in the pulmonary circulation overriding the passive increase in PCWP. As demonstrated in Table 3, mPAP as well as PCWP were elevated before dialysis compared with afterwards, whereas TPG did not differ significantly. Moreover, in all CKD-precapillary-PH patients the stable “out-of-proportion" TPG suggests a precapillary component in addition to the fluid overload before dialysis. Therefore the elevated TPG might point towards CKD- precapillary-PH already at the time before hemodialysis. However, we propose that RHC should be performed after dialysis to unmask precapillary PH. The Dana Point classification assigns PH in patients with CKD to Group 5, i.e. all of the patients in the present study primarily belong into this group [8], [9]. However, based on the present post-dialysis hemodynamics, a re-grouping of some CKD patients with precapillary PH into Group 1 at least is to be discussed in upcoming guidelines. Certainly there is need for more data, i.e. if histopathological changes of the here-described cohort are similar to those patients in Dana Point group 1 (PAH). We explicitly do not want to encourage CKD patients with a precapillary PH to be treated with specific PAH drugs, as there is no knowledge about the efficacy of those therapies in this cohort.

There are notable limitations of this single-center study. Each patient was only measured once invasively. Especially in the hemodialysis cohort it would be possible that RHC would have led to different results over the time caused by different fluid overload/dry weight. However, we think that our invasive approach is new and more catheterizations cannot be enforced in this patients' cohort. We did not obtain data measuring extracellular fluid (ECF). Another limitation is the measurement of CI by direct Fick method. Due to arteriovenous fistula, the product of the Fick formula and therefore CI and PVR might be changed. However, there is no influence on PAP and PCWP.

Treatment regimens were not assessed. Therefore, the question how best to treat patients with CKD and PH/PAH remains unresolved. Although information on specific PAH treatment or clinical outcome would be of interest, it would necessitate a controlled, prospective study analyzing clinical end points with specific PAH treatment and requiring a follow-up period of several years and the recruitment of a large patient cohort. With the exception of isolated case reports [22], the efficacy of current medical therapeutics for PAH such as prostanoids, endothelin receptor antagonists and phosphodiesterase-5 inhibitors have not been studied in CKD patients.

In conclusion, this study provides evidence that precapillary PH is a common co-morbidity in CKD patients on dialysis. End-stage kidney disease and/or hemodialysis rather than the renal insufficiency itself seems to be the main determining risk factor for developing precapillary PH. Diagnostic RHC should be performed after dialysis to diferentiate precapillary from postcapillary PH.
